# Visualization of Moisture Content, Reducing Sugars, and Chewiness in Bread During Oral Processing Based on Hyperspectral Imaging Technology

**DOI:** 10.3390/foods13223589

**Published:** 2024-11-10

**Authors:** Xiaoyu Tian, Qin Fang, Xiaorui Zhang, Shanshan Yu, Chunxia Dai, Xingyi Huang

**Affiliations:** 1 School of Food and Biological Engineering, Jiangsu University, Xuefu Road 301, Zhenjiang 212013, China; tianxy@ujs.edu.cn (X.T.); fangqin200011@163.com (Q.F.); 15751003723@163.com (X.Z.); yushannie_lucky@163.com (S.Y.); 2 School of Electrical and Information Engineering, Jiangsu University, Xuefu Road 301, Zhenjiang 212013, China; txdcx@126.com

**Keywords:** bread, oral processing, chewiness, hyperspectral imaging, visual distribution

## Abstract

This study evaluated the differences in oral processing and texture perception of breads with varying compositions. The research investigated the dynamic changes in moisture content (MC), reducing sugars (RSs), and chewiness of the bolus formed from white bread (B0) and 50% whole-wheat bread (B50) during oral processing. Hyperspectral imaging (HSI) combined with chemometric methods was used to establish quantitative prediction models for MC, RSs, and chewiness, and to create visual distribution maps of these parameters. The results showed that B0 had a higher moisture content and a faster hydration rate than B50 during the initial stages of oral processing, indicating greater hydrophilicity and ease of saliva wetting. Additionally, the uniformity of moisture distribution in the bolus of B0 was higher than that of B50. B50 exhibited significantly lower RSs content and poorer distribution uniformity compared to B0. The primary differences in chewiness between the two types of bread were observed during the early stages of oral processing, with B50 requiring more chewing effort initially. This study demonstrated that HSI technology can effectively monitor and elucidate the compositional changes in food particles during oral processing, providing new insights into bread texture perception and offering a scientific basis for improving bread processing and texture.

## 1. Introduction

Bread is a significant staple food that is widely consumed around the world, contributing substantially to the daily nutritional intake across various populations [1]. The formulation of bread, including the type of flour, water content, yeast, and other additives, is crucial in determining the bread’s texture, flavor, and nutritional qualities [2]. Wheat flour, as the primary ingredient in bread, is characterized by its high starch content, leading to a generally high glycemic index (GI) [3]. Adding a certain proportion of whole-wheat flour can significantly reduce the glycemic index (GI), but at the same time, due to its high dietary fiber content, it also brings about an unpleasant taste, thereby reducing consumer acceptability [4,5]. Therefore, conducting a scientific and quantitative assessment of the physicochemical and textural characteristics of bread is of great significance for optimizing the bread-making process.

The primary methods for sensory evaluation of bread typically involve both qualitative and quantitative approaches, such as chemical analysis [6], texture profile analysis (TPA) [7,8], and a 1–10 scoring system [9]. These methods are employed to assess various properties of bread, including hardness, chewiness, flavor, and overall acceptability to consumers [4]. While these methods provide certain sensory information, they are primarily based on static product evaluation and typically do not involve the dynamic changes that occur during mastication. However, sensory evaluation is a psychological and physiological process that relies on human interaction with food, namely oral processing [10]. During the oral processing of bread, it undergoes complex processing such as being crushed by teeth, moistened by saliva, and mixed by the tongue, gradually forming a food bolus [11,12]. The process of food forming a bolus is the dynamic and real perception process of consumers’ experience with the food [13,14]. Therefore, closely monitoring the oral processing of bread and accurately assessing its dynamic changes are essential for understanding the sensory experience of consumers.

The sensory experience of bread is a complex process involving the physical changes of food structure, oral physiology, and the dynamic interaction of personal perception. The changes in texture, flavor, and taste of bread during the oral processing stage are important aspects of the sensory evaluation of bread. Researchers have regarded oral processing as a key method for evaluating the sensory quality of bread and the interaction with the consumer’s perception. Puerta et al. [15] studied the particle size of the bread bolus formed after three chews, the bolus characteristics at the swallowing point, and oral activities, and pointed out that the breakdown and bolus formation behavior of bread in the mouth can fully explain the dynamic perception of bread texture. Pu et al. [16] investigated the dynamic release and perception of key taste compounds in bread during the oral processing stage. Gao et al. [11] focused not only on the physical structure of bread but also delved into the sensory perception of bread during the oral processing stage, including texture, saltiness, and aroma. Mosca et al. [17] analyzed the oral processing behavior of both a commercial gluten-free (GF) bread and its gluten-containing (GC) counterpart by determining oral processing parameters using video recordings. Faridi Esfanjani and Mohebbi [18] used oral processing as an important evaluation method to understand how the texture, structure, and composition of bread influence the sensory perception and acceptance of low salt bread. Li et al. [19] examined the impact of pumpkin preparation on the oral processing and sensory texture perception of bread during mastication.

It is noteworthy that the bolus plays a significant role in the oral processing of bread, yet the depth of research on it requires further enhancement. Current research predominantly examines the bolus at the point of swallowing, with less attention given to its evolving characteristics throughout the oral processing. In addition, the bolus is a heterogeneous composite, and studying it as a whole may overlook many detailed pieces of information, such as the variations in its composition and texture distribution. Therefore, it is necessary to delve into the detailed information of bolus formation during oral processing to analyze its characteristics more comprehensively.

Hyperspectral imaging (HSI), a fusion of near-infrared spectroscopy and imaging, enables nondestructive analysis of heterogeneous samples. It also utilizes distribution maps to illustrate the spatial distribution of target compounds within these samples, making it a valuable tool for visualizing the composition of diverse components in food and agricultural products. HSI technology has been utilized for the prediction and visualization of food components, such as the soluble solids content and pH in kiwifruit [20], microstructural changes and moisture distribution in dry-cured pork [21], particle size distribution in matcha [22], total polyphenols distribution in tea [23], total acidity and hardness of red globe grapes [24], moisture and anthocyanins content distribution in purple sweet potato [25], ingredients distribution in pizza [26], and moisture distribution in beef [27]. However, there have been no reports on the application of HSI technology for the visualization of component distribution within food boluses during oral processing.

The objectives of this study are (1) to investigate the dynamic changes in the moisture content, reducing sugars content, and chewiness of boluses formed from two types of bread with different compositions during the oral processing; (2) to establish quantitative prediction models for the moisture content, reducing sugars content, and chewiness of bread boluses based on hyperspectral information; (3) to use these quantitative prediction models to create visual distribution maps that characterize the evolution of hydration, enzymatic breakdown, and textural properties of bread during oral processing, followed by a correlation analysis.

## 2. Materials and Methods

**Figure 1:**
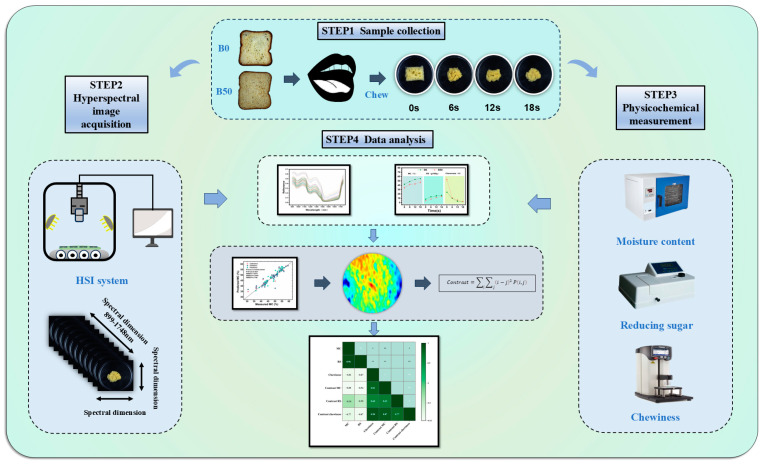
Schematic representation of the oral processing of bread, measurement of physical and chemical properties, and hyperspectral images acquisition.

A schematic diagram of the oral processing of bread, the measurement of physical and chemical properties, and hyperspectral images acquisition is shown in Figure 1.

### 2.1. Materials and Chemicals

The main ingredients for bread preparation included high-gluten wheat flour and whole-wheat flour, both obtained from Xinghua Cereal and Oil Foodstuff Co., Ltd. (Qingdao, China). Active dry yeast and butter were sourced from Angel Yeast Co., Ltd. (Yichang, China), while pure milk was provided by Bright Dairy & Food Co., Ltd. (Shanghai, China). Eggs and white granulated sugar were purchased locally from Jimailong Supermarket (Zhenjiang, China). All chemicals used, including glucose, phenol, sodium tartrate, 3,5-dinitrosalicylic acid, sodium bisulfite, and sodium hydroxide, were of analytical grade and supplied by Sinopharm Group Chemical Reagent Co., Ltd. (Shanghai, China).

### 2.2. Bread Making

The bread was prepared using a standard protocol with a fully automatic bread machine, which included kneading, fermentation, and baking stages. The entire process took approximately 2 h, baking time 60 min, baking temperature 180–200 °C, as described by Shimada and Yoshimura [28]. After baking, the loaves were removed from the machine, allowed to cool at room temperature for 1 h, and then sliced into uniform 1-cm-thick pieces using a commercial bread slicer. The sliced bread was then packaged in airtight containers to preserve freshness. The bread samples, labeled B0 and B50, varied in whole-wheat flour content, with B0 containing no whole-wheat flour and B50 containing 50% whole-wheat flour. The detailed formulations for each bread type are provided in Table 1.

### 2.3. Bread Collection

The oral processing of bread was studied at Jiangsu University’s College of Food and Biological Engineering. Eight volunteers (four female, four male), aged 20 to 38, were recruited based on strict selection criteria: all were in good health, had no history of dental disease, and exhibited normal chewing, salivary secretion, olfactory, and gustatory functions. None of the participants were smokers. The experiment was conducted daily between 9 AM and 11 AM under controlled environmental conditions (temperature: 22 ± 2 °C; humidity: 50 ± 10%) to ensure a standardized testing environment. Volunteers were instructed to refrain from consuming alcohol and eating spicy or irritating foods for 2 h prior to the experiment to minimize external influences on saliva composition and flow.

Following the methods outlined by Pu et al. [16], the oral processing of bread was divided into four intervals: 0, 6, 12, and 18 s. Each participant chewed each bread sample at a frequency of two cycles per second, with each sample weighing 3.5 g (±0.2 g). Participants were instructed to expectorate the chewed bolus at the designated time intervals for further analysis. Three replicates were collected for each time interval, and the order of sample collection was randomized to reduce potential biases.

### 2.4. Physiochemical Indicators Analysis of Bolus

#### 2.4.1. Measurement of Moisture Content

The moisture content (MC) of the bolus was determined by first measuring and recording the mass of the expectorated bolus. The bolus was then placed in a drying oven maintained at a constant temperature of 105 °C until it reached a constant weight. The MC was calculated as the percentage of weight loss during drying relative to the initial weight after oral processing, using the following formula:

(1) $$MC=\frac{m_{1}-m_{2}}{m_{2}}$$

where $m_{1}$ refers to the mass of chewed bolus, $m_{2}$ refers to the mass of dried bolus.

#### 2.4.2. Measurement of Reducing Sugars Content

The content of reducing sugars (RSs) was determined using the 3,5-dinitrosalicylic acid (DNS) method. A glucose standard curve was established by preparing a series of glucose solutions with known concentrations and measuring their absorbance at 540 nm using a 752N ultraviolet-visible spectrophotometer (Jinghua Technology Instrument Co., Ltd., Shanghai, China).

The bolus samples were extracted, mixed with the DNS reagent, and incubated at 50 °C for 10 min to develop color. The absorbance of the resulting solution was then measured at 540 nm. The concentration of RSs in the bolus samples was calculated from the standard curve.

#### 2.4.3. Measurement of Chewiness

Texture profile analysis (TPA) of the bread bolus was conducted using a TMS-Pro texture analyzer (Food Technology Co., Ltd., Rockland, MA, USA). TPA tests were conducted immediately after chewing to minimize the impact of salivary enzymes on the bread mass, with the bread bolus being sequentially chewed and tested for different chewing times. The chewed bolus was gently flattened with a glass rod on the sample platform to ensure uniform contact with the P50 probe. The compression test was carried out with pre-test, test, and post-test speeds of 2.0, 1.0, and 1 mm/s, respectively. The strain displacement was set to 5 mm, with a trigger force of 5 g. Each bolus was compressed for 5 s to measure the chewiness parameter, which was recorded in triplicate to ensure result reliability.

### 2.5. Hyperspectral Image Acquisition and Processing

The hyperspectral imaging system, consisting of an ImSpector V10E camera (Spectral Imaging Ltd., Oulu, Finland), a Fiber-Lite DC-950 illuminator (Dolan-Jenner Industries Inc., Boxborough, MA, USA), an SC30021A automated translation stage (Zolix Instruments Co., Ltd., Beijing, China), and a computer with P4P800-X HSI analysis 15.0.1 software (Asus Computer Co., Ltd., Taiwan, China), was used for spectral data acquisition. The system was preheated for 30 min to stabilize baseline drift, as recommended by Zhang et al. [29]. Spectral scanning was performed over a range of 899 to 1748 nm, covering 512 wavelengths. To ensure image clarity and prevent distortion, the sample stage movement speed was set to 5.3 cm/s. The bread bolus sample was placed in a glass Petri dish for testing.

To mitigate potential noise from factors such as dark current and uneven illumination, black and white correction was applied using the following formula:

(2) $$R=\frac{I-B}{W-B}$$

where *R* is the calibrated image, *I* is the raw image, and *B* and *W* are the black and white reference images, respectively [30,31].

After calibration, a 100 × 100 pixel region of interest (ROI) was selected from the center of the sample. The spectral values within the ROI were averaged to obtain a representative spectral value for each sample [32]. To enhance spectral reproducibility and reduce noise, the collected spectra were preprocessed using normalization (N), Gaussian filtering (GF), and Savitzky–Golay smoothing (SG) methods, as recommended by Cheng et al. [33,34].

Partial least squares regression (PLSR) was employed to determine the linear relationship between spectral data and physicochemical properties, effectively avoiding multicollinearity and enhancing prediction stability [35]. Additionally, principal component regression (PCR) was applied to project the data onto principal components, reducing data redundancy and improving model generalization [36].

### 2.6. Visualization of MC, RS, and Chewiness

Each pixel within the sample’s region of interest (ROI) was input into the optimal prediction model, and the distribution of key physicochemical indicators across all pixels was visualized using pseudo-color data processing.

### 2.7. Contrast Analysis

Contrast refers to the moment of inertia near the main diagonal of the Gray Level Co-occurrence Matrix (GLCM). It reflects the distribution of values within the matrix and indicates both the clarity of the image and the depth of texture grooves [37]. Contrast is calculated from four directions—0°, 45°, 90°, and 135°—using the GLCM using the specific formula as follows:

(3) $$contrast=\Sigma_{i}\Sigma_{j}{\left(i-j\right)}^{2}P\left(i,j\right)$$

where *i* and *j* are the row and column indices of the GLCM, and *P*(*i*, *j*) represents the (*i*, *j*)th entry in the normalized GLCM.

### 2.8. Statistical Analysis

Spectral preprocessing and feature selection were performed using Unscrambler X 10.4 software (Camo Analytics, Oslo, Norway). MATLAB 2023a (MathWorks, Natick, MA, USA) was utilized for model building and data analysis. Graphs were generated with Origin2023a (OriginLab Corp., Northampton, MA, USA), and ENVI 5.3 (Harris Geospatial Solutions, Boulder, CO, USA) was used to extract reflective spectra from hyperspectral images. To analyze correlations among datasets, the Spearman correlation matrix was calculated using SPSS 19.0 (IBM Corporation, NY, USA), selected for its non-parametric approach, which is well-suited to the characteristics of our data.

## 3. Results and Discussion

### 3.1. Bread Moisture Content, Reducing Sugars, and Chewiness

**Figure 2:**
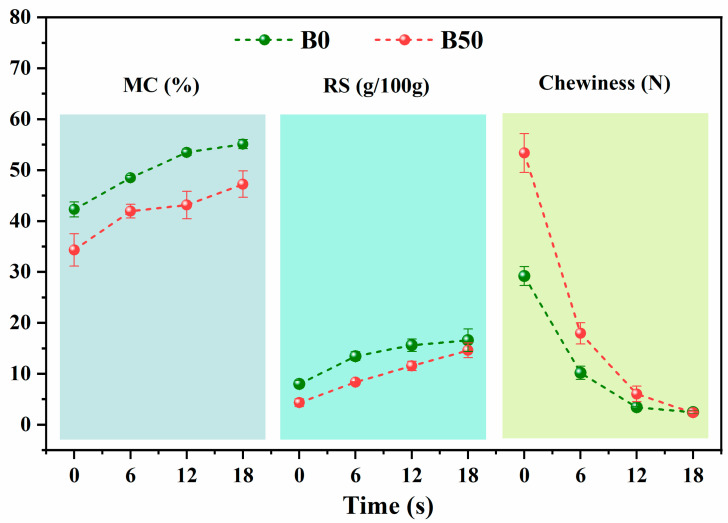
Changes in moisture content (MC), reducing sugars (RSs), and chewiness during bread oral processing.

Figure 2 illustrates the changes in moisture content (MC), reducing sugars (RSs) content, and chewiness of white bread (B0) and 50% whole-wheat bread (B50) during oral processing. As oral processing time increased to 18 s, the MC of B0 rose from an initial 42.28% to 55.10%, while B50’s MC increased from 34.33% to 47.24%. Notably, both the initial MC and hydration rate during chewing were higher for white bread (B0) compared to B50, suggesting that B0 has a greater affinity for water and is more readily wetted by saliva. Tebben et al. [38] reported that water-insoluble fiber constituents inhibit the hydration of gluten proteins and starch. The addition of 50% whole-wheat flour in B50, which contains cellulose and lignin from the bran, may limit saliva penetration and distribution, making the bread more resistant to moistening during oral processing. Similar results were found by Rashed et al. [39], who observed that bread with 50% oat flour exhibited a dry, chewy texture, likely due to its high dietary fiber content.

RSs contribute to sweetness perception during bread consumption. As shown in Figure 2, the RSs content in both B0 and B50 bread gradually increases with oral processing time, indicating that salivary α-amylase breaks down starch into shorter sugar chains, such as maltose and glucose, which raises the RSs levels. Overall, B50 has a lower RSs content than B0. The higher gluten and fiber content in B50 results in a coarser texture, which may impede the even distribution of saliva on the bread surface, thereby reducing the effectiveness of salivary amylase [40] and slowing the conversion of starch into reducing sugars [41]. Pentikainen et al. [42] found that bread composition plays a key role in the release of compounds during oral processing, with tri-, tetra-, and monosaccharides being released to a greater extent from masticated wheat bread compared to masticated rye bread. Similarly, Wang et al. [43] reported that adding 40% quinoa flour significantly reduced starch digestibility and the maximum digestion rate by 17%, indicating that incorporating fiber-rich quinoa flour can lower the starch digestibility of bread.

Chewiness, reflecting the energy needed to break down food into a swallowable consistency, is determined by the combined effects of hardness, cohesiveness, and elasticity [44]. As a key indicator of food texture, chewiness influences consumer acceptance and preference. The chewiness value of B50 significantly decreased from 53.36 N to 17.93 N, whereas B0 experienced a more substantial drop, from 29.16 N to 10.17 N. Despite the decrease, B50 still demonstrated a markedly higher chewiness compared to B0. However, when the chewing time increased to 18 s, the chewiness of B50 dropped to 2.44 N, and the difference between the two breads gradually decreased, eventually becoming negligible. Majzoobi et al. [45] reported that bread containing 30% whole-oat flour had more than double the chewiness of white bread. Similarly, Rashed et al. [39] found that bread with 50% oat flour exhibited increased chewiness, likely due to the dietary fiber in oat flour negatively affecting dough formation, resistance to extension, and extensibility, leading to a denser and firmer texture. Feng et al. [46] reached similar conclusions, noting that the addition of whole quinoa flour significantly increased the chewiness of steamed bread. These findings highlight significant differences between B0 and B50 bread in moisture content, reducing sugars content, and chewiness, particularly during the early stages of oral processing.

### 3.2. Spectral Characteristics and Preprocessing

**Figure 3:**
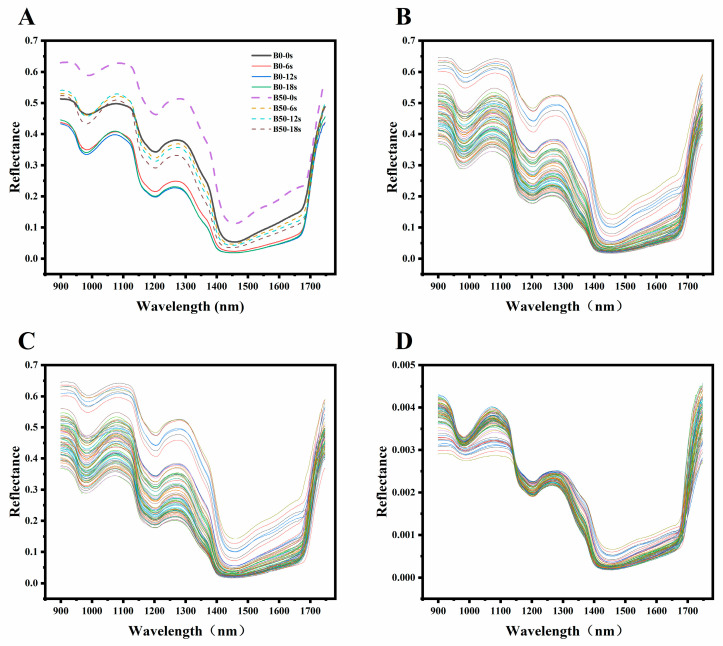
Spectral profile of bread at different stages of oral processing. (**A**) Average raw spectral at different stages of oral processing. (**B**) Spectral preprocessed by SG algorithm. (**C**) Spectral preprocessed by GF algorithm. (**D**) Spectral preprocessed by normalize algorithm.

As shown in Figure 3, during the different stages of oral processing, the reflectance spectrum of the bread bolus indeed exhibits noticeable changes. With the increase of oral processing time, the reflectance of the bread bolus gradually decreases. This change may be attributed to alterations in the bolus’s internal composition and content during oral processing, including the release of moisture, sugars, and the breakdown of starch, all of which can impact spectral reflectance.

The absorption peaks at specific wavelengths provide crucial information about the chemical composition of the bolus. The absorption peak near 960 nm may be associated with the second overtone vibration of O-H, which is prevalent in water and carbohydrates [47,48]. This indicates that moisture and carbohydrates within the bolus may undergo changes during the oral processing, leading to variations in the reflected spectral profile. The intense absorption peak at 1200 nm is attributed to the second overtone of the C-H stretching vibration, typically associated with the fat content in the sample [49,50]. Changes in this absorption peak may reflect the release or structural alterations of fat components within the bolus, which is crucial for understanding the sensory characteristics of bread during oral processing. The third intense absorption peak near 1450 nm can be ascribed to the first overtone of the O-H stretching vibration [51,52]. Variations in this peak may indicate changes in moisture content within the bolus, as the O-H bond vibration is closely linked to moisture content.

Additionally, the N-H groups in proteins exhibit specific vibrational patterns in the near-infrared spectrum, which are closely related to the secondary and tertiary structures of the proteins. For instance, the various N-H vibrations detected in the 1015 to 1026 nm range are a significant indicator of protein presence. The signals related to N-H vibrations in the 1500 to 1520 nm range may reveal specific arrangements and interactions of amino acids within the protein [53,54]. These characteristic absorption peaks not only reflect changes in the protein’s organizational structure but also explain the differences in textural properties of the food bolus.

### 3.3. Establishment of Regression Model

The performance of partial least squares regression (PLSR) and principal component regression (PCR) models for predicting key quality indicators during bread oral processing was systematically evaluated. The models were developed using full-wavelength spectral data as the input (X variables) and the corresponding measured MC, RS, and chewiness as the output (Y variables). As shown in Table 2, the results indicate that PLSR models, especially those preprocessed with Savitzky–Golay (SG) smoothing, outperformed their PCR counterparts across all parameters.

Specifically, in the prediction of MC, the PLSR model with SG smoothing demonstrated the highest predictive accuracy, with a coefficient of determination, $R_{p}^{2}$, of 0.8898 and a root mean square error of prediction (RMSEP) of 2.0526. This indicates a strong linear relationship between the predicted and actual MC values. Similarly, for the prediction of RSs content, the PLSR model following the SG smoothing treatment showed the best prediction accuracy, yielding an $R_{p}^{2}$ of 0.8783 and an RMSEP of 1.4936. The chewiness was best predicted by the PLSR model post-SG smoothing, achieving an $R_{p}^{2}$ of 0.9012 and a RMSEP of 4.6410. Figure 3 provides a visual representation of the models’ predictive performance, exhibiting a pronounced linear correlation between the measured and predicted values for MC, RS, and chewiness, indicating the models’ exceptional predictive capabilities.

**Figure 4:**
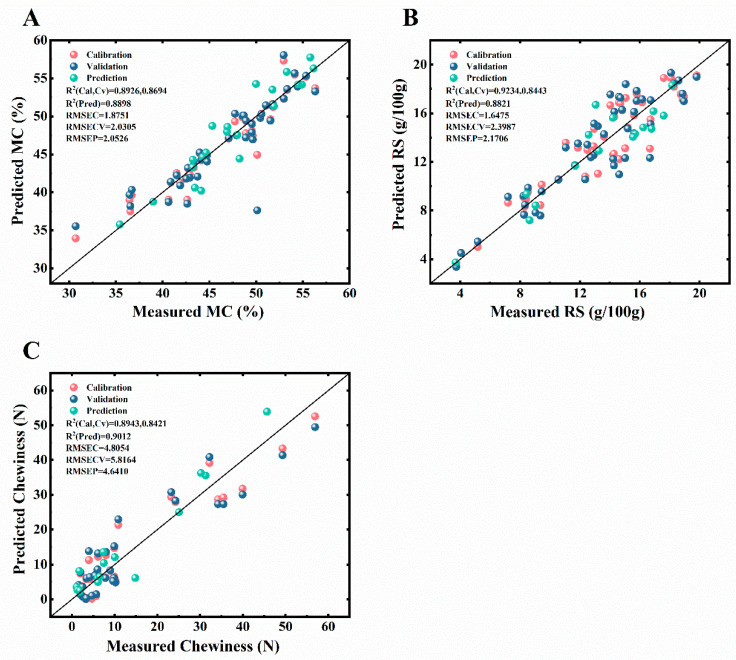
Correlation between predicted and measured values for moisture content (MC), reducing sugars (RSs), and chewiness. The solid line represents the ideal prediction line, while the dashed line indicates the linear regression fit. (**A**) MC prediction, (**B**) RSs prediction, and (**C**) chewiness prediction.

In the prediction of MC, the PLSR model with Savitzky–Golay smoothing demonstrated the highest predictive accuracy, with a coefficient of determination $R_{p}^{2}$ of 0.8898 and a root mean square error of prediction (RMSEP) of 2.0526. This reflects a strong linear relationship between the predicted and actual MC values. Similarly, for RSs content, the PLSR model with SG smoothing showed the best accuracy, achieving an $R_{p}^{2}$ of 0.8783 and a RMSEP of 1.4936. The chewiness prediction was also most accurate with the PLSR model after SG smoothing, with an $R_{p}^{2}$ of 0.9012 and an RMSEP of 4.6410. Figure 4 visually illustrates the models’ predictive performance, revealing a pronounced linear correlation between the measured and predicted values for MC, RSs, and chewiness, underscoring the models’ strong predictive capabilities.

### 3.4. Visual Distribution

**Figure 5:**
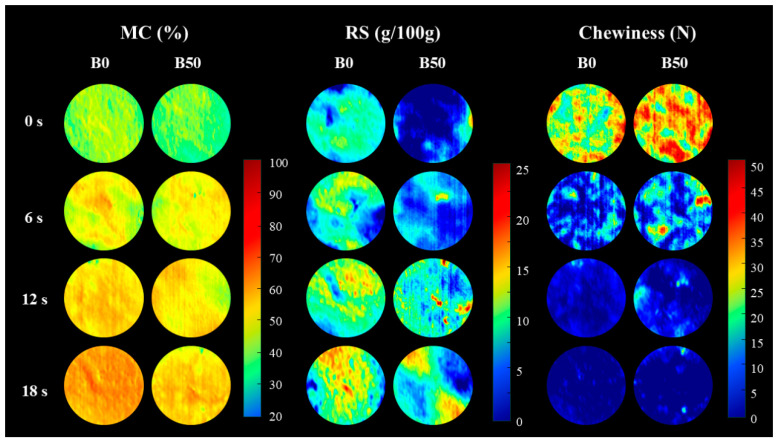
Distribution map of moisture content (MC), reducing sugars (RSs), and chewiness of two types of bread at different stages of oral processing.

Figure 5 shows the visual distribution of MC, RSs, and chewiness of the bread pellets during the oral processing. The levels of MC, RSs, and chewiness in the representative samples are depicted using a color bar on the right side of the image, with colors ranging from red to blue. The cooler colors (blue and green) indicate lower levels of MC, RSs, and chewiness in the processed samples, while the warmer colors (red, orange, and yellow) represent higher levels of these attributes.

The initial MC distribution of the bread is uniform. However, by oral processing of 6 s, the moisture distribution becomes increasingly uneven. As oral processing continues to 12 and 18 s, the distribution gradually becomes more uniform again. During oral processing, the bread is repeatedly crushed and mixed with saliva in the mouth. In the early phase (0–6 s), due to insufficient oral processing, saliva primarily concentrates on the edges of the minimally crushed bread. As oral processing occurs, the bread particles become smaller and mix more thoroughly with saliva, resulting in a more even distribution of moisture. Additionally, saliva secretion is relatively low and uneven during the early stages of oral processing, leading to some areas of the food not being adequately wetted, causing uneven moisture distribution [16,55]. Moreover, the uniformity of moisture distribution in B50 bread is lower than in B0, likely due to the higher dietary fiber content in B50, which reduces its hydrophilicity [38].

As oral processing time increases, the RSs content distribution map gradually shifts from blue to yellow and red, with the yellow and red areas in B0 bread being noticeably larger than those in B50 bread. This suggests that during oral processing, B0 bread has a higher RSs content and better homogeneity than B50 bread. During oral processing, bread undergoes both physical breakdown and enzymatic hydrolysis, with the food being fractured multiple times. Salivary amylase then continuously degrades starch through a cyclic process of salivation and mixing [56,57]. Differences in the particle size of the food bolus, resulting from the composition of the bread [15,58], affect the contact surface between salivary amylase and the bread. Consequently, B50 bread, which contains more fiber, has significantly lower RSs content and poorer homogeneity compared to B0 bread.

Chewiness refers to the amount of work required to masticate food to a swallowable state, as illustrated by the distribution changes in Figure 5. As oral processing time increases, the chewiness of both types of bread decreases rapidly. By 12 s, the chewiness of both breads converges, indicating that they reach the swallowing point at this time. The main difference in chewiness between the two breads occurs within the first 6 s of oral processing. After 6 s, some areas of B50 bread still appear red, suggesting that these regions remain rough and firm. This implies that during shorter oral processing times, saliva may not fully penetrate and soften the coarse fibers in B50 bread, resulting in a rough and firm texture in certain areas [11]. Generally, the pore structure and closed porosity of bread affect the amount of work required for chewing [59]. Bread made with whole-wheat flour typically requires more chewing effort than white bread [39,60], with some areas feeling firmer during the initial stages of chewing. While measurements of MC, RSs, and chewiness are typically taken on the entire food sample, a more detailed analysis of the visual distribution of these factors can offer deeper insights into food quality and texture.

### 3.5. Correlation Analysis

**Figure 6:**
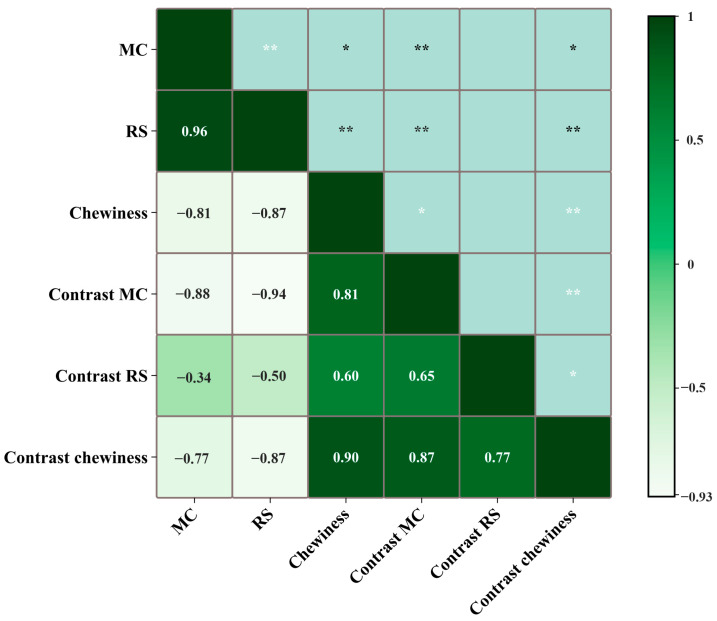
Correlation matrix of moisture content (MC), reducing sugars (RSs), chewiness, contrast MC, contrast RSs, and contrast chewiness during bread oral processing. * *p* ≤ 0.05, ** *p* ≤ 0.01.

The correlation matrix for MC, RSs, chewiness, and their contrast is shown in Figure 6. During oral processing, significant correlations are observed between MC, RSs, and chewiness. Specifically, MC and RSs are positively correlated (*p* < 0.01), MC and chewiness are negatively correlated (*p* < 0.05), and RSs and chewiness are negatively correlated (*p* < 0.01). This indicates that during oral processing, MC and RSs levels follow a similar trend, while chewiness follows an opposite trend. The oral processing of bread involves the gradual formation of a smooth, soft, and swallowable food bolus. This process is accompanied by the infiltration of saliva, amylase hydrolysis, and a reduction in both hardness and chewiness.

Additionally, Figure 6 demonstrates a significant negative correlation (*p* < 0.01) between the uniformity of MC and chewiness. In contrast, no significant correlation is observed between the uniformity of MC and RSs. As depicted in Figure 5, the uniformity of both MC and chewiness initially increases and then decreases as oral processing occurs, whereas the uniformity of RSs decreases continuously over time. This trend may be attributed to the fact that amylase hydrolysis commences only after the bread is moistened by saliva, a process that necessitates both time and space to manifest its effects. This aligns with the observation that it takes several seconds to perceive a noticeable sweetness [16]. In summary, the correlation matrix provides the patterns of physicochemical and textural changes that occur during the oral processing of bread, elucidating the mechanisms underlying the alterations in bread bolus properties throughout the oral processing.

## 4. Conclusions

In this study, two types of bread with different formulations were prepared, and the variations in moisture content (MC), reducing sugars (RSs), and chewiness during oral processing were investigated. By utilizing HSI coupled with chemometric approaches, predictive models for these three key parameters were developed. The PLSR-SG model achieved the highest predictive accuracy for MC, RSs, and chewiness, with $R_{p}^{2}$ values of 0.8898, 0.8783, and 0.9012, respectively. The corresponding RMSEP values were 2.0526, 1.4936, and 4.6410. Additionally, distribution maps based on the optimal models were created to visually assess the uniformity of these indicators during oral processing. The results indicated that white bread (B0) and 50% whole-wheat bread (B50) exhibited significantly different moisture contents and absorption rates during the initial stages of oral processing. B0 had a higher moisture content and faster absorption rate, suggesting greater hydrophilicity and saliva wetting ability. Moreover, the moisture distribution in the bolus of B0 was more uniform than that of B50. In contrast, B50 had significantly lower RSs content and poorer distribution uniformity. As oral processing progressed, both types of bread showed increases in MC and RSs, while chewiness decreased. The chewiness difference between B0 and B50 was more pronounced in the early stages of oral processing but converged as processing time increased.

This study demonstrated the value of examining both the visual distribution characteristics and conventional measurements of key indicators for a deeper understanding of bread quality and consumer sensory experience. Hyperspectral imaging technology was highlighted as a powerful tool for analyzing physicochemical changes in food during oral processing. It not only enables the quantitative prediction of food components but also allows for the visualization of material changes through distribution maps, thereby offering insights into food structure and properties that can aid in food development and quality control.

## Figures and Tables

**Table 1:** The detailed formulation of breads.

Bread Type	Formulation
B0	High-gluten wheat flour 50%, white granulated sugar 6%, pure milk 40%, salt 0.5%, butter 3%, active dry yeast 0.5%, one egg
B50	High-gluten wheat flour 25%, whole-wheat flour 25%, white granulated sugar 6%, pure milk 40%, salt 0.5%, butter 3%, active dry yeast 0.5%, one egg

**Table 2:** Performance of moisture content (MC), reducing sugars (RSs), and chewiness (CH) during bread oral processing under different models.

Models	Type	$R_{C}^{2}$	RMSEC	$R_{CV}^{2}$	RMSECV	$R_{P}^{2}$	RMSEP
SG-PLSR	MC	0.9498	1.2817	0.8890	1.9302	0.8549	2.3551
	RSs	0.9106	1.3931	0.8654	1.8316	0.8783	1.4936
	CH	0.8943	4.8045	0.8421	5.8164	0.9012	4.6410
GF-PLSR	MC	0.9476	1.3102	0.8754	2.0831	0.8509	2.3880
	RSs	0.9109	1.4409	0.8374	2.1118	0.8374	1.7266
	CH	0.8944	4.8031	0.8470	5.5786	0.9009	4.6472
N-PLSR	MC	0.9435	1.3605	0.8472	2.9007	0.8461	2.4259
	RSs	0.8834	1.6979	0.8302	2.0787	0.8135	1.8491
	CH	0.8872	4.9634	0.8104	6.6499	0.8968	4.7423
SG-PCR	MC	0.9431	1.3642	0.8684	2.0913	0.8558	2.3479
	RSs	0.8856	1.6323	0.8438	2.1145	0.8123	1.8549
	CH	0.8923	4.8514	0.8737	5.6536	0.8990	4.6919
GF-PCR	MC	0.9430	1.3657	0.8822	2.0189	0.8552	2.3528
	RSs	0.8860	1.6296	0.7977	2.2002	0.8120	1.8564
	CH	0.8923	4.8506	0.8366	5.6550	0.8987	4.6980
N-PCR	MC	0.8926	1.8751	0.8694	2.0305	0.8898	2.0526
	RSs	0.8635	1.7832	0.7830	2.2496	0.8034	1.8985
	CH	0.8582	5.5652	0.7884	6.9983	0.8805	5.1040

SG—Savitzky–Golay; GF—Gauss filter; N—normalization; MC—moisture content; RSs—reducing sugars; CH—chewiness; PLSR—partial least squares regression; PCR—principal component regression; $R_{C}^{2}$—coefficient of determination with calibration; $R_{CV}^{2}$—coefficient of determination with cross-validation; $R_{P}^{2}$—coefficient of determination with prediction; RMSEC—root mean square error of calibration; RMSECV—root mean square error of cross-validation; RMSEP—root mean square error of prediction.

## Data Availability

The original contributions presented in the study are included in the article, further inquiries can be directed to the corresponding author.
